# Saharan Dust Events in the Dust Belt -Canary Islands- and the Observed Association with in-Hospital Mortality of Patients with Heart Failure

**DOI:** 10.3390/jcm9020376

**Published:** 2020-01-30

**Authors:** Alberto Dominguez-Rodriguez, Néstor Baez-Ferrer, Sergio Rodríguez, Pablo Avanzas, Pedro Abreu-Gonzalez, Enric Terradellas, Emilio Cuevas, Sara Basart, Ernest Werner

**Affiliations:** 1 Department of Cardiology, Hospital Universitario de Canarias, 38320 La Cuesta, Santa Cruz de Tenerife, Spain; adrvdg@hotmail.com (A.D.-R.); nestor.baez@hotmail.com (N.B.-F.); 2 Faculty of Health Sciences, Universidad Europea de Canarias, Calle Inocencio García 1, 38300 La Orotava, Santa Cruz de Tenerife, Spain; 3 Centro de Investigación Biomédica en Red Enfermedades Cardiovaculares (CIBERCV), 28029 Madrid, Spain; 4 Experimental Stations of Arid Zones, EEZA CSIC, Carretera del Sacramento, 04120 La Cañada de San Urbano, Almería, Spain; 5 Instituto de Productos Naturales y Agrobiología, IPNA CSIC, Avenida Astrofisico Francisco Sánchez 3, La Laguna 38206, Tenerife, Spain; 6 Department of Cardiology, Central University Hospital of Asturias, Avenida de Roma, 33011 Oviedo, Spain; avanzas@gmail.com; 7 Department of Medicine, University of Oviedo, Julián Clavería 6, Campus del Cristo, 33006 Oviedo, Spain; 8 Health Research Institute of the Principality of Asturias, Avenida de Roma, 33011 Oviedo, Spain; 9 Department of Basic Medical Sciences, University of La Laguna, Santa María Soledad, 38200 La Cuesta, Santa Cruz de Tenerife, Spain; pabreugonzalez@gmail.com (P.A.-G.); ewernerh@aemet.es (E.W.); 10 SDS-WAS Regional Centre, AEMET, Arquitecte Sert 1, 08005 Barcelona, Spain; enric.terradellas@gmail.com; 11 Izaña Atmospheric Research Centre, AEMET, La Marina 20, 38001 Santa Cruz de Tenerife, Spain; ecuevasa@aemet.es; 12 Barcelona Supercomputing Centre, Jordi Girona 29-31, 08034 Barcelona, Spain; sara.basart@bsc.es

**Keywords:** Saharan dust, in-hospital mortality, heart failure, dust belt

## Abstract

Recent studies have found increases in the cardiovascular mortality rates during poor air quality events due to outbreaks of desert dust. In Tenerife, we collected (2014–2017) data in 829 patients admitted with a heart failure diagnosis in the Emergency Department of the University Hospital of the Canaries. In this region, concentrations of PM_10_ and PM_2.5_ are usually low (~20 and 10 µg/m^3^), but they increase to 360 and 115 μg/m^3^, respectively, during Saharan dust events. By using statistical tools (including multivariable logistic regressions), we compared in-hospital mortality of patients with heart failure and exposure to PM_10_ and PM_2.5_ during dust and no-dust events. We found that 86% of in-hospital heart failure mortality cases occurred during Saharan dust episodes that resulted in PM_10_ > 50 µg/m^3^ (interquartile range: 71–96 µg/m^3^). A multivariate analysis showed that, after adjusting for other covariates, exposure to Saharan dust events associated with PM_10_ > 50 µg/m^3^ was an independent predictor of heart failure in-hospital mortality (OR = 2.79, 95% CI (1.066–7.332), *p* = 0.03). In conclusion, this study demonstrates that exposure to high Saharan dust concentrations is independently associated with in-hospital mortality in patients with heart failure.

## 1. Introduction

Health effects linked to the exposure of ambient air pollutants is a major environmental issue and results in ~3 million deaths a year, mostly due to ischaemic heart disease (~40%), stroke (~40%), chronic obstructive pulmonary disease (~11%), lung cancer (~6%) and acute lower respiratory infections (~3%, mostly in children), according to the World Health Organization [1]. In urban areas, the population is mostly exposed to combustion-linked reactive gases (NO_x_, SO_2_ and O_3_) and aerosols (i.e., particulate matter (PM)) typically containing black carbon, organics, sulphate, nitrate, ammonium, road-dust and trace metals. After this epidemiological evidence [1], the medical community started to develop so-called “environmental cardiology” studies focusing (i) on understanding the pathophysiological mechanisms by which combustion PM prompts atherosclerosis and ischemic heart disease, and (ii) on identifying the role of gene-environment interactions and the pathways involved in the oxidative stress generated in vascular inflammation [2,3,4]. These studies showed that inhalation of diesel exhaust soot particles promoted proatherogenic genes in vascular endothelial cells, whereas ambient ultrafine particles lead to prooxidant and proinflammatory effects prompting atherosclerosis [2,5,6]. These studies contributed to identify the profile of the persons especially susceptible to combustion PM and who should take medical preventive actions [7].

**Figure 1:**
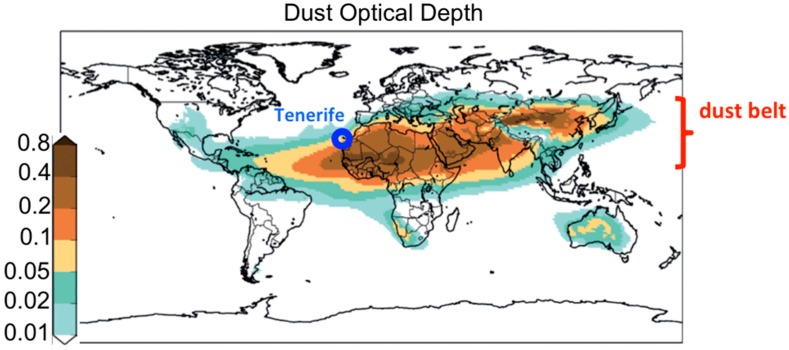
Global Dust Optical Depth highlighting the location of Tenerife and of the Dust Belt (WHO, 2017).

More recently, the scientific community started to pay attention to the health effects linked to the inhalation of desert dust aerosols [8,9], especially cardiovascular disease [10,11,12]. Most desert dust sources are located in the so-called “dust belt” (Figure 1), which expands through North Africa, the Middle East and to China [13]. Dust from North Africa is mostly exported to the Atlantic, resulting in frequent dust concentrations within the ranges (i) thousands µg/m^3^ in western North Africa [14] and (ii) tens to hundreds (µg/m^3^) in the Canary Islands [15] and Cape Verde [16]. Episodically, dust is exported northward across the Mediterranean, typically resulting in dust concentrations of about tens of µg/m^3^ in southern Europe (typically 10–60 µg/m^3^) [17,18]. Epidemiological studies performed in cities of southern Europe found increases in cardiovascular mortality during Saharan dust events [19]. The biological mechanism by which dust is associated with cardiovascular mortality remains unknown [11,12].

There are two clear gaps in the studies of health effects linked to dust exposure. First, there is a need to develop studies in the population living near major dust sources. After a literature review, De Longueville et al. [20] concluded that there was an imbalance between the location of the major dust sources (North Africa to Middle East) and most studied regions (southern Europe and East Asia). The highest dust concentration occurs in western North Africa, and no studies on the health effects of dust, in practice, have been performed in the region. Second, most studies have been epidemiological, associating cardiovascular mortality to dust; however, the specific heart disease involved in that cardiovascular mortality statistic is still unknown (is dust prompting angina, acute myocardial infarction, acute coronary syndrome or heart failure (HF), or several of them?); this information is vital so hospital emergency departments can prepare for when a severe dust event is forecasted. This study attempts to contribute to fill these two gaps.

The results presented here are part of a set of studies designed to identify the pathophysiological mechanisms by which exposure to desert dust aerosols blowing in the ambient air influences the prognosis of cardiovascular disease. Here we focused on HF, a chronic and progressive condition by which the heart is unable to maintain the pumping needed to provide the body’s needs for blood and oxygen [21]. HF is the main cause of emergencies and hospitalization in patients over the age of 65, and there is a need to identify the factors that may have led to suffer such HF events [22]. The objective is to assess if exposure to desert dust aerosols influences in-hospital mortality in patients suffering from HF.

## 2. Materials and Methods

### 2.1. Study Region

Tenerife (Figure 1) has an abrupt orography, with a mountain ridge which runs from the centre of the island (Las Cañadas, 2400 m.a.s.l., base of El Teide volcano, 3718 m.a.s.l.) toward the northeast (Anaga, reaching 1024 m.a.s.l.). Meteorology is dominated by the North Atlantic anticyclone, which prompts trade winds to blow. The main sources of air pollutants are road traffic and the Candelaria and Granadilla fuel-oil power plants. The oil refinery in Santa Cruz definitively ceased its refining activities in 2013. Levels of air pollutants are rather low, compared to continental regions of Europe [23,24].

### 2.2. Dust Events

We characterised the Saharan dust events using PM_x_ measurements and dust modelling. Concentrations of PM_10_ and PM_2.5_ (PM with an aerodynamic diameter <10 and 2.5 microns, respectively) and reactive gases are measured by the Air Quality Network of the Canary Islands by using harmonized standard methods in the European Union. We determined and analysed daily averaged values. We initially analysed data of PM_x_ collected at three sites of Tenerife, placed in the south (El Rio rural site), the northeast (Tena Artigas urban site in Santa Cruz de Tenerife) and the north (Balsa Zamora rural site, near Los Realejos).

The surface dust concentrations and dust optical depth provided by the multimodel median prediction (https://sds-was.aemet.es/forecast-products/dust-forecasts) of the WMO SDSWAS (World Meteorological Organization’s Sand and Dust Storm Warning Advisory and Assessment System) was used to identify dust events [25]. This ensemble product is generated from the forecasts provided by twelve dust prediction models (by using the poor-man approach) [26]: BSC-DREAM8b, CAMS, DREAM8-NMME-MACC, NMMB/BSC-Dust, MetUM, GEOS-5, NGAC, RegCM4-EMA, DREAMABOL, NOA-WRF-CHEM, SILAM and LOTOS-EUROS. The evaluation of this dust forecast for the Canary Islands was performed by García-Castrillo and Terradellas [27].

### 2.3. Medical Data

This study was based on medical data collected in the Emergency Department of the University Hospital of the Canary Islands, Tenerife, in patients admitted with the diagnosis of HF. We studied the period from 2014 to 2017, and 829 patients were admitted with this diagnosis. The following demographics, clinical variables, HF precipitating factors, and in-hospital treatments were collected by an independent researcher (Table 1):

- demography—age and gender;
- cardiovascular risk factors—dichotomous variable reporting if the patient had hypertension, was a smoker, had diabetes mellitus or hypercholesterolemia;
- medical history—dichotomous variable on previous HF episodes, previous chronic ischemic heart disease (IHD), atrial fibrillation or chronic obstructive pulmonary disease (COPD);
- biochemistry—levels of hemoglobin (g/dL), brain natriuretic peptide (BNP: pg/mL) and sodium (mg/dL) were determined by analysis of blood samples;
- clinical data—data on the left ventricular ejection fraction (LVEF: %), hospital stay (d) and Charlson index;
- HF precipitating factors—therapeutic non-compliance, rapid atrial fibrillation, infections and unknown precipitating factors;
- in-hospital treatment—dichotomous variable reporting if patients received treatment with furosemide, spironolactone / eplerenone, beta blockers, angiotensin-converting enzyme inhibitor (ACEI) or angiotensin II receptor antagonists (ARA-II) during the period they stayed in the hospital.

### 2.4. Statistical Analysis

The objective was to study the in-hospital mortality, more specifically, to identify how the independent variables were associated with in-hospital HF mortality (dependent variable). As independent variables we used the medical data described above and data of PM_x_ and dust events, more specifically, the daily mean PM_10_, PM_2.5_ and PM_2.5–10_ concentrations and the occurrence of a Saharan dust resulting in a (24 h average) PM_10_ concentration >50 µg/m^3^ (dichotomous variable). The data of PM_x_ collected at El Rio station, directly exposed to the Saharan dust events arriving to Tenerife, were used for this analysis. To compare quantitative variables, we used the Mann–Whitney U test and the Student’s *t* test. To determine the association between qualitative (dichotomous) variables, the chi-square test or Fischer’s exact test were used. The variable “Saharan dust event with PM_10_ 50 μg/m^3^” (24 h average) was categorized as a dichotomous variable (1 or 0). Multivariable logistic regression analysis was carried out to determine the variables associated with the presence of in-hospital mortality. Statistical analysis was performed with the SPSS program, version 20 (SPSS Inc., Armonk, NY, USA). This methodology is typically used in these types of studies [28].

## 3. Results

### 3.1. Dust Events

**Figure 2:**
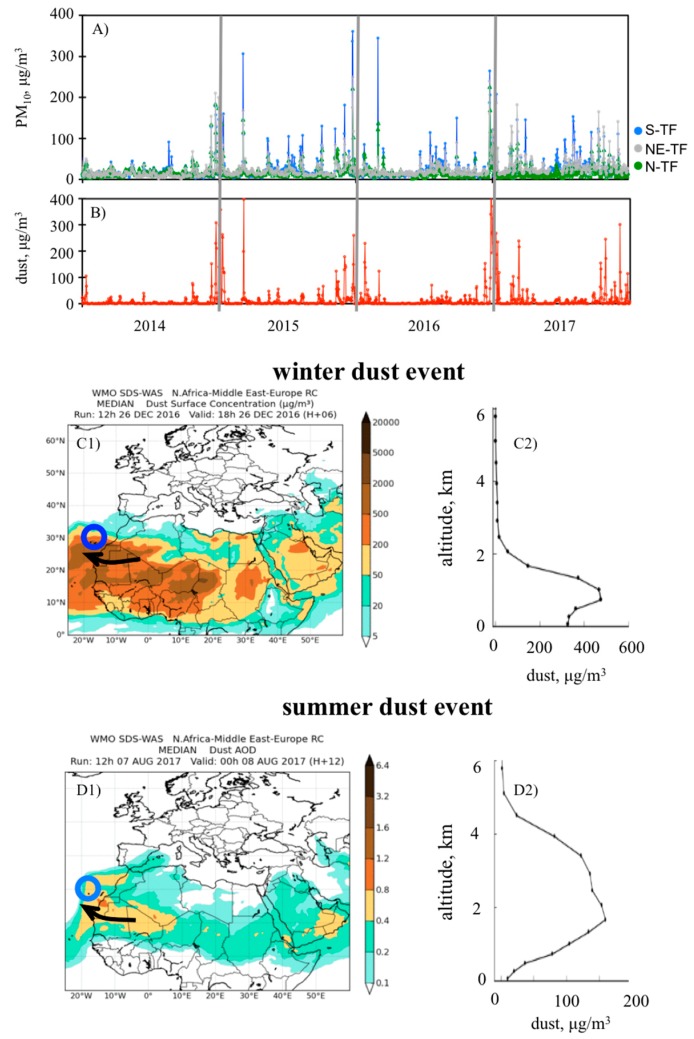
Time series of daily mean values of (**A**) PM_10_ measured at three sites of Tenerife (South, S-TF; Northeast, NE-TF; and North, N-TF) and of (**B**) surface dust concentrations provided by the World Meteorological Organization’s Sand and Dust Storm Warning Advisory and Assessment System (WMO SDSWAS). Example of a typical dust event of winter (26-Dec-2016): surface dust concentrations (**C1**) and vertical dust profile in Tenerife (**C2**). Example of a typical dust event of summer (08-Aug-2017): dust optical depth (**D1**) and vertical dust profile in Tenerife (**D2**). Blue circle highlights the location of Tenerife.

As far as we know, this is among the first studies on dust and cardiovascular disease performed in the dust belt (Figure 1), so we first illustrated the huge impacts of Saharan desert dust on air quality. Figure 2A shows the daily average concentrations of PM_10_ on Tenerife Island during the study period (2014–2017), whereas Figure 2B shows the dust concentrations at the surface level provided by WMO SDSWAS modelling [27].

**Figure 3:**
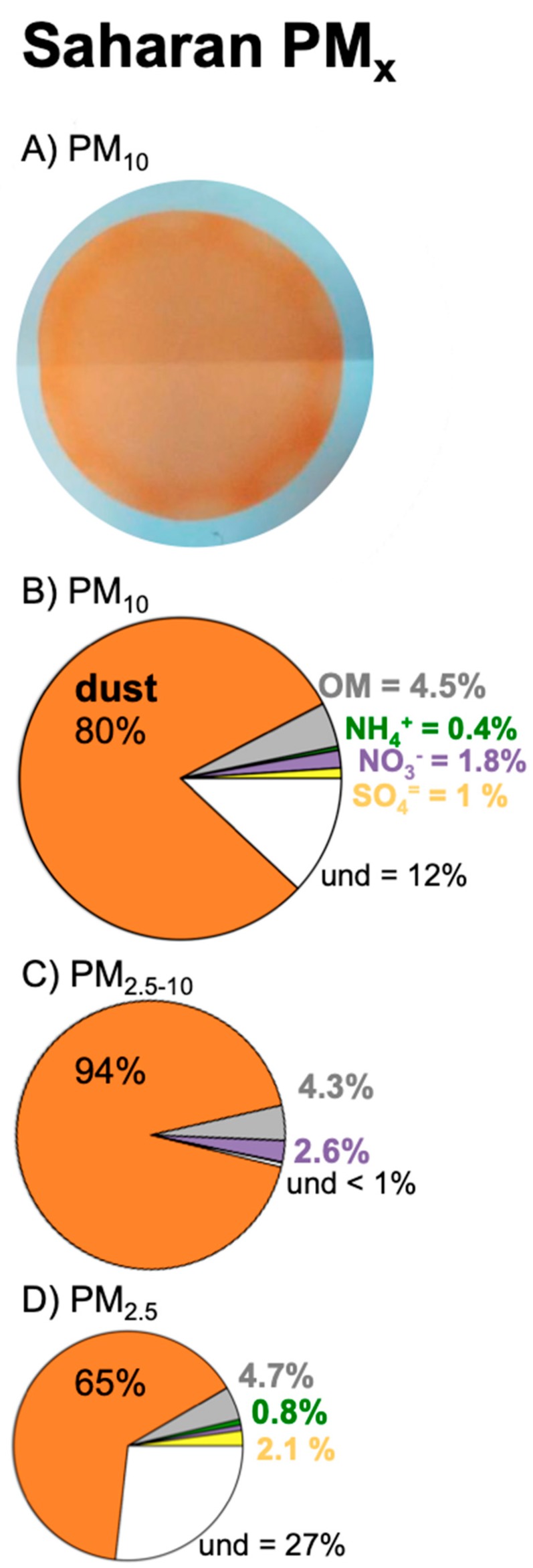
(**A**) Picture of a PM_10_ sample collected from the ambient air (at the rate of 30 m^3^/h during 8 h) during a Saharan dust event. The samples were collected in a filter, and the ochre colour of the samples evidenced the desert dust nature; the outer white layer is the blank part of the filter (no sample). (**B**–**D**) Mean bulk composition of PM_x_ in Tenerife during Saharan dust events according to García et al. [30], highlighting the contributions of desert dust, organic matter (OM), ammonium (NH_4_^+^), nitrate (NO_3_^−^), sulphate (SO_4_^=^) and the undetermined fraction (und). The und fraction is mostly water attached to the aerosols.

Background levels of PM_10_ were usually low, with an annual 50th percentile within the range 13–17 µg/m^3^ and annual mean values with the range 18–24 µg/m^3^ at the three sites (south, north and northeast) of Tenerife plotted in Figure 2A. Previous studies [29] found that the composition of PM_10_ associated with these background levels was dominated by sea salt (~30%), fuel oil combustion (25%), vehicle exhaust (12%) and dust (12%). During Saharan dust events, PM_10_ concentrations increased from the background level to values within the ranges 35–50 µg/m^3^ (during “moderate” events), 50–100 µg/m^3^ (“intense”) and 100–400 µg/m^3^ (“very intense”; Figure 2A). In these cases, PM_10_ was, by far, constituted by mineral dust [29,30] (see the typical ochre colour and the chemical composition of PM_10_ samples during these events in Figure 3).

At low altitudes (near sea level), winter dust events were very frequently associated with higher dust and, consequently, PM_10_ concentrations than those of summer dust events; the measured PM_10_ (Figure 2A) and dust models (Figure 2B) reached values within the range 100–400 µg/m^3^ between November and March and within 30–100 µg/m^3^ in summertime. These differences between the winter and the summer dust events were due to the altitude at which the dusty Saharan Air Layer (SAL) arrived to Tenerife. In winter, dust transport occurs at low altitudes (approximately <700 m.a.s.l. [31]). In summertime, the dusty SAL tends to flow at higher altitudes (~500 to 5000 m.a.s.l. [15,32]); impacts at ground occur when the SAL shifts downward [33]. Examples of winter and summer dust events are shown in Figure 2C,D, including the dust vertical profile. Because the core path of the SAL regularly occurs south of the Canary Islands, dusty air arrives to Tenerife under southeast airflow conditions and, consequently, PM_10_ concentrations are frequently higher in southern than in northern Tenerife (the latter also affected by the shielding effect of the orography).

### 3.2. Impact of Dust on In-Hospital Mortality of Patients with HF

We focused on the impact on HF in-hospital mortality of the Saharan dust events with PM_10_ concentrations (24 h average) higher than 50 µg/m^3^, which is the threshold value for PM_10_ recommended in the guidelines of the World Health Organization. These events were identified using WMO SDSWAS modelling (Figure 2C,D). During the study period, the threshold of 50 µg/m^3^ was exceeded on 124 days, all them associated with Saharan dust episodes.

During the study period (2014–2017), 829 patients were admitted in the Emergency Department of the University Hospital of the Canary Islands with the diagnosis of HF. From this group, a total of 49 patients expired (5.9%), whereas 780 survived, which is close to the average values in Spain (9.4% for people over 45 years [22]). By applying the tests and the multivariable logistic regression analysis described in the methodology, we analysed the association of HF in-hospital mortality (dependent variable) with the demographics, cardiovascular risk, medical history, biochemistry, clinic data, HF precipitating factors, in-hospital treatments of each patient and dust events as independent variables. The results are shown in Table 1. The two groups (i.e., HF mortality and HF no mortality) showed no significant differences in terms of the demographics, clinical characteristics, HF precipitating factors, and in-hospital treatment variables (*p* > 0.05). Factors such as smoking, suffering from diabetes mellitus or hypertension, or previous COPD or HF did not differentiate deceased patients from patients that survived. However, a huge difference was obtained in terms of exposure to Saharan dust (*p* < 0.0001; Table 1). Eighty-six percent of deaths (42/49 cases) registered (2014-2017) in the HF mortality group occurred during Saharan dust episodes that resulted in PM_10_ concentrations higher than 50 μg/m^3^; more specifically, this group was exposed to PM_10_, PM_2.5_ and PM_2.5-10_ within the interquartile ranges 71–96, 23–36 and 37–69 μg/m^3^, respectively. The group of patients that suffered no HF mortality were mostly exposed to lower PM_x_ levels, with a Q3 equal to 26 μg/m^3^ for PM_10_, 12 μg/m^3^ for PM_2.5_ and 14 μg/m^3^ for PM_2.5-10_. However, about 41% of these patients were exposed to PM_10_ > 50 μg/m^3^, that is, most of these admissions occurred during a few intense dust events. The multivariate analysis we performed in the present study showed, after adjusting for other covariates, that exposure to Saharan dust events with PM_10_ concentrations higher than 50 μg/m^3^ was an independent predictor of in-hospital mortality in patients with HF (OR = 2.79, 95% CI (1.066–7.332), *p* = 0.03).

## 4. Discussion

Recent epidemiological studies found an association between cardiovascular mortality and Saharan dust episodes affecting southern Europe. The current study is part of a set of investigations designed to identify the pathophysiological mechanisms by which exposure to desert dust aerosols prompts cardiovascular disease. A previous study showed that exposure to dust events with PM_10_ > 50 μg/m^3^, age and key comorbidity factors (as hypertension and diabetes mellitus) were precipitating factors for hospital admission due to HF [10]. In this new study we found an association between exposure to high concentrations of Saharan desert dust and in-hospital mortality of patients with HF; more specifically, we found that 86% of in-hospital HF mortality cases (2014–2017) were registered during Saharan dust episodes that resulted in PM_10_ concentrations higher than 50 μg/m^3^. 

The most intense dust events mostly occur in winter, Nov to Mar (Figure 2B), so this is the period when HF mortality due to dust may occur in our study region. In other regions, dust seasons occur in different seasons (e.g. spring in the Middle East and in Asia). Thus, because the results of this study can most probably be extrapolated to other regions, and because we try to address this topic from a comprehensive point of view, we have not emphasised seasonal evolution. In other words, HF mortality due to dust will be enhanced during dust seasons, whenever they are. In summary, exposure to dust concentrations of PM_10_ > 50 μg/m^3^ (guideline value of the World Health Organization) exacerbates HF and may result in deaths. These results are especially relevant for the dust belt, where the population is exposed to dust concentrations within the hundreds to thousands µg/m^3^ range.

The results of this and previous studies performed by our group point out that exposure to desert dust aerosols and combustion aerosols probably results in different types of cardiovascular diseases. In our study, we found an association between desert dust aerosols and HF, whereas previous studies [34] found an association between combustion soot (black carbon aerosols) and ischemic heart diseases, including acute coronary syndrome. These results suggest that coarse dust particles exacerbate HF, whereas very small combustion particles tend to deposit in the coronary arteries. Consistently, epidemiological studies found that mortality was associated with fine (PM_2.5_) particles during non-Saharan dust days and with coarse (PM_2.5–10_) particles during Saharan dust days [35]. Further complementary studies to understand the pathophysiological mechanisms are needed.

## 5. Conclusions

Exposure to Saharan dust events associated with PM_10_ concentrations >50 μg/m^3^ is an independent predictor of HF in-hospital mortality.

## Figures and Tables

**Table 1:** Baseline characteristics of the study population as a function of in-hospital mortality.

	HF Mortality (*n* = 49)	HF no Mortality (*n* = 780)	*p*-Value
Age, year	73.5 ± 2	73.8 ± 1.5	0.25
Female sex	13 (26.5)	220 (28.2)	0.80
**Cardiovascular risk factors**			
Hypertension	25 (51)	500 (64.1)	0.06
Smoking	24 (49)	413 (52.9)	0.58
Diabetes mellitus	20 (40.8)	339 (43.5)	0.71
Hypercholesterolemia	30 (61.2)	454 (58.2)	0.67
**Medical history**			
Previous HF episode	33 (67.3)	462 (59.2)	0.26
Previous chronic IHD	8 (16.3)	179 (22.9)	0.28
Atrial fibrillation	10 (20.4)	242 (31)	0.11
COPD	12 (24.5)	126 (16.2)	0.12
**Biochemistry**			
Hemoglobin (g/dL)	11.95 ± 1.65	11.73 ± 1.73	0.37
BNP (pg/mL)	1295.8 (937.8–1769)	1339.6 (925.6-1961.8)	0.29
Sodium (mg/dL)	138.31 ± 2.88	138.32 ± 3.15	0.98
**Clinical data**			
LVEF (%)	49.47 ± 12.40	50.86 ± 11.04	0.39
Hospital stay (d)	10.59 ± 3.67	10.37 ± 3.77	0.66
Charlson index	4.73 ± 0.78	4.71 ± 0.74	0.84
**HF precipitating factors**			0.73
Therapeutic non-compliance	2 (4)	59 (7.9)	
Rapid atrial fibrillation	25 (52.1)	365 (48.8)	
Infections	12 (25)	208 (27.7)	
Unknown precipitating factors	14 (28.5)	144 (18.4)	
**In-hospital treatment**			
Furosemide	49 (100)	780 (100)	-
Spironolactone / Eplerenone	31 (63.3)	499 (64)	0.92
Beta blockers	32 (65.3)	462 (59.5)	0.41
ACEI	36 (73.5)	540 (69.2)	0.53
ARA-II	13 (26.5)	240 (30.8)	0.53
Number of patients exposed to PM_10_ ≥ 50 μg/m^3^ during Saharan dust events	42 (85.7)	318 (40.8)	<0.0001
PM_10_ (μg/m^3^)	84.7 (71.5–95.8)	15.3 (9.7–26.4)	<0.0001
PM_2.5_ (μg/m^3^)	29.9 (23.3–36.1)	6.9 (5.6–12.5)	<0.0001
PM_2.5-10_ (μg/m^3^)	57.6 (37.5–68.8)	8.3 (5.6–13.9)	<0.0001

Continuous values are expressed as mean ± standard deviation or median (Q1: Q3), categorical values with n (%). ACEI, angiotensin-converting enzyme inhibitor; ARA II, angiotensin II receptor antagonists; BNP, brain natriuretic peptide; COPD, chronic obstructive pulmonary disease; HF, heart failure; IHD, ischemic heart disease; LVEF, left ventricular ejection fraction; PM, particulate matter.
